# Supplementary material for: Light programmable micro/nanomotors with optically tunable in-phase electric polarization
Source: Nat Commun. 2019 Nov 21;10:5275. doi: 10.1038/s41467-019-13255-6 (PMC6872749; doi:10.1038/s41467-019-13255-6)
Supplement: Supplementary file 1 — Supplementary Info [file 41467_2019_13255_MOESM1_ESM.docx]

**Supplementary Information**

**Light Programmable Micro/Nanomotors with Optically Tunable In-Phase Electric Polarization**

Liang et al.

Description of Additional Supplementary Files:

1. File Name: Supplementary Movie 1

Description: This movie demonstrates the tracking of nanowires with our custom software. The user manipulates the greyscale threshold to obtain the best tracking of nanowires.

1. File Name: Supplementary Movie 2

Description: Light switches the alignment direction of a nanowire from perpendicular to parallel to an electric field. Here, a nanowire is placed in the parallel microelectrodes, to which a 15 Vpp and 750 kHz waveform is applied. A laser beam of 111 $mW/cm^{2}$ is used to switch the nanowire alignment direction to be parallel to the field. When the laser is off, the nanowire restores to perpendicular alignment.

1. File Name: Supplementary Movie 3

Description: A nanowire rotates back and forth to signal “HELLO WORLD” in Morse code, demonstrating a unique method of communication. The nanowire is placed in the parallel microelectrodes with an electric field of 750 kHz at 25 Vpp so that it aligns perpendicular to the field. Laser light is toggled programmatically by a DMD to switch the nanowire in two directions.

1. File Name: Supplementary Movie 4

Description: Rotation modes at different AC voltages with light. Here, a nanowire in the quadruple microelectrodes is driven by an electric field of $f_{1}=100 \mathrm{kHz}$ rotating at $f_{2}=1.49 \mathrm{Hz}$, where the voltage applied to the microelectrodes starts with 12 Vpp and then changes to 20 Vpp. The nanowire is exposed to 318 $mW/cm^{2}$ laser.

1. File Name: Supplementary Movie 5

Rotation modes at different AC voltages with light. A nanowire is placed in an electric field of $f_{1}=100 \mathrm{kHz}$ rotating at $f_{2}=1.5 \mathrm{Hz}$ in the quadruple microelectrodes, and constantly exposed to 318 $mW/cm^{2}$ laser. The voltage applied to the microelectrodes is scanned from 2 Vpp to 28 Vpp.

1. File Name: Supplementary Movie 6

Description: Light effect on the rotation of a nanowire at different AC frequencies. A nanowire (in the quadruple microelectrodes) is driven by an electric field of $f_{1}=100 \mathrm{kHz}$ at 30 Vpp rotating at a frequency ($f_{2})$ from 1.5 Hz to 23.8 Hz. A laser of 318 $mW/cm^{2}$ is toggled on and off to switch the rotation modes of the nanowire. Above 8.9 Hz, the nanowire cannot rotate when the laser is off.

1. File Name: Supplementary Movie 7

Description: A nanowire rotates synchronously with an electric field up to maximum speed, after which it exhibits out-phase rotation before stopping. Here, a nanowire is placed in a rotating electric field of $f_{1}=100 \mathrm{kHz}$ at 30 Vpp in quadruple microelectrodes, and constantly exposed to laser light of 318 $mW/cm^{2}$. The electric field rotation frequency $f_{2}$ is scanned from 1.5 Hz to 26.8 Hz.

1. File Name: Supplementary Movie 8

Description: Demonstration of the utilization of light to control the operation mode of a nanomotor: a nanowire rotates continuously at synchronous speed with an electric field (“in-phase”) with laser illumination, but barely twitches (“out-phase”) without light. Here, a nanowire (in the quadruple microelectrodes) is driven by an electric field of $f_{1}=100 \mathrm{kHz}$ rotating at $f_{2}=2.98 \mathrm{Hz}$ at 25 Vpp. A laser of 127 $mW/cm^{2}$ is toggled on and off to switch the alignment of the nanowire.

1. File Name: Supplementary Movie 9

Description: Demonstration of the utilization of light to control the operation mode of multiple nanomotors. Many nanowires are driven by an electric field of $f_{1}=100 \mathrm{kHz}$ rotating at $f_{2}=2.98 \mathrm{Hz}$ at 30 Vpp. A laser of 318 $mW/cm^{2}$ is toggled on and off to control the switching of the nanowires.

1. File Name: Supplementary Movie 10

Description: Individually controlled nanowire motors with DMD-generated laser spots. Here, a DMD generates two laser spots of 50 $mW/cm^{2}$, which are each exposed to one of two nanowires (in the quadruple microelectrodes). The nanowires are driven by an electric field of $f_{1}=100 \mathrm{kHz}$ rotating at $f_{2}=1.49\mathrm{Hz}$ at 13 Vpp. The laser spots are then turned on and off independently, which can control one motor without affecting the other.

1. File Name: Supplementary Movie 11

Description: Operation of a nanowire to designated angular positions as a stepper motor. Here, the angle of a nanowire (in the quadruple microelectrodes) is controlled by tuning the direction of an electric field of 100 kHz at 10 Vpp with illumination of 127 $mW/cm^{2}$ laser.

1. File Name: Supplementary Data 1

Description: This file (“Morse.dat”) contains data of the position and angle of a nanowire versus time as it is stimulated by an intermittent laser to communicate words in Morse code (see Supplementary Movie 3). Interpret and plot this data to see the signals in Morse code by placing this file in the same folder of the MATLAB script “interpreter.m” before executing the script.

1. File Name: Supplementary Data 2

Description: This is a MATLAB script (“interpreter.m”) that is used to interpret and plot the nanowire motion data in the “Morse.dat” file to show its Morse-code signals. Place the “morse.dat” file in the same folder before running this script.

**Supplementary Note 1: Kramers-Kronig Relation: suggest but cannot predict**

We observed the light induced change in the imaginary part of polarizability as shown in our previous study. From what the K-K relation suggests, the real part of the polarizability could also be changed by light. Based on this, we find it will be interesting to experimentally study the effect of light on the real part of polarization of semiconductor nanowires.

However, at this stage, we are not able to directly calculate the real part imaginary part of polarizability from the previously experimentally measured imaginary part because of the following obstacles:

First, in our previous work, we measured the imaginary part of nanowire polarizability from the experiment of electro-rotation. The electro-rotation experiment is conducted by applying a circularly polarized AC electric field (direction of the field is rotating at the same frequency of oscillation). The electro-rotation speed is directly proportional to $Im(\alpha_{\parallel}+\alpha_{\perp})$. However, in this work, the electro-alignment experiment is based on the real-part of polarization, in which the alignment rate is proportional to $Re(\alpha_{\parallel}-\alpha_{\perp})$. According to Kramers-Kronig relation, $\mathrm{Im}\left( \alpha_{\parallel} \right)$ and $Re(\alpha_{\parallel})$ are correlated, $\mathrm{Im}\left( \alpha_{\perp} \right)$ and $Re(\alpha_{\perp})$ are correlated. Although $\alpha_{\perp}$ is usually much smaller compared to $\alpha_{\parallel}$ (Supplementary Figure 1A, C), we are not able to strictly determine $Re(\alpha_{\parallel}-\alpha_{\perp})$ from the experimentally obtained $Im(\alpha_{\parallel}+\alpha_{\perp})$ via the K-K relation.

Here, we mentioned the suggestion of the K-K relation because, for nanowire with long aspect ratio, the polarizability along the longitudinal axis is usually much larger than that along the transverse axis, i.e. $\mathrm{Im}\left( \alpha_{\parallel} \right)\gg Im\left( \alpha_{\perp} \right)$and $\mathrm{Re}\left( \alpha_{\parallel} \right)\gg Re(\alpha_{\perp})$, which are satisfied at most frequencies (not including specific frequencies when $\mathrm{Im}\left( \alpha_{\parallel} \right)$and $\mathrm{Re}\left( \alpha_{\parallel} \right)$ are close to zero, e.g. as that shown in Supplementary Figure 1A and C). Therefore, the K-K relation could suggest the light tunability of the real-part of polarizability from the experimental observed light tunable imaginary-part of polarizability.

Another obstacle in strictly utilizing the K-K relation comes from the experimental aspect. To implement the K-K relation, the integral contour is the entire upper half-plane, which means, the value of one part, either imaginary or real, has to be known over all frequencies. Experimentally, only a limited range of frequencies can be measured, and the common way to implement the K-K relation is to interpolate or extrapolate for the missing data via certain approximation. For our case, at high frequency limit, both real and imaginary parts of polarizability approach zero, however, at low frequencies *i.e.* below 1 kHz, the behavior of polarizability remains unmeasurable because of the co-existence of other electrokinetic effects: first, at low frequencies, the electrical double layer at the electrode-solution interface will screen most voltage for effective electric field applied on a nanowire; second, at low frequencies, AC electric field will induce strong AC osmosis flow in aqueous solution; last, electrohydrolysis reaction starts at lower voltages with reduced AC frequency. All of them conceal the motion of nanowire from the polarization effect. Due to the above, we are not able to directly measure the electric polarization generated motions at low frequencies to quantitatively determine the real-part and imaginary parts of polarizability from each other by using the K-K relation.

**Supplementary Note 2: Experimental Apparatus**

In all our experiments, as discussed in the methods section of the main paper, silicon nanowires are suspended in DI water inside a PDMS liquid cell placed on the microelectrodes (either parallel or quadruple). A custom 4-output function generator sets the voltage bias on each microelectrode contact. A 532-nm diode-pumped solid-state laser (Thorlabs) with controlled intensity, is projected onto the Si nanowires through the cover glass on top of the liquid cell, which is also illuminated dimly by a custom LED diffused white light source of about 500 lx in order to minimize variability from background light. A camera (Basler acA1300-200um), operating up to $1280\times1024$ pixels and 1000 frames per second (FPS), images the nanowires from below after the light going through a long pass filter that blocks the laser, which could otherwise damage the camera. A custom software, based on standard computer vision algorithms in C++, tracks the positions and angles of nanowires over time for later data analysis.

The camera continuously feeds images to this program, which uses the OpenCV computer vision library to recognize nanowires. Specifically, it thresholds, blurs, and extracts contours from each 8-bit grayscale image frame. Contours within a manually-set area and aspect ratio limits are set to trace nanowires, so their position and angle can be recorded, along with a unique identification number. Nanowires are tracked between frames by matching contours to their nearest neighbors with some hysteresis to account for blurred frames and other problems. The program saves the movie and records the nanowire locations and angles in real time. The entire camera-computer system regularly achieves 1000 FPS in a small image area, e.g. 200x200 px, and approximately 200 FPS at a full resolution of 1280x1024. A demonstration of the system in tracking nanowires can be found in Supplementary Movie 1.

In addition to tracking nanowires, this control and image analysis program interfaces with the custom 4-output function generator creating the microelectrode bias voltages. The function generator is capable of creating arbitrary +/-15 V (30 Vpp) AC outputs or DC bias at up to 1 MHz. This is necessary for, *e.g.*, E-field rotation, for which each of the four quadruple microelectrodes must be driven by a product of two sine waves (that is, high and low frequencies for alignment and rotation, respectively). The control program may reset the output phase of the function generator at low rotation frequencies (< 100 Hz) while simultaneously keeping accurate time in order to track subsequent E-field phase changes as used to measure load angle.

Finally, the laser may be reflected off a digital light projection (DLP) system using a digital micromirror device (DMD) (specifically, a DLi 4130 high-speed development kit using Texas Instrument’s DLP7000 DMD chip) in order to modulate the beam before getting in the microscope optics. This allows the laser to be toggled programmatically, as utilized in the demonstration of Morse code light stimuli (Supplementary Movie 3), and even to be masked into multiple, individually controlled spots, allowing rotation of singled-out nanowires (Supplementary Movie 10).

**Supplementary Note 3: Alignment Rate Measurement and Analysis**

The induced electric dipole moment ($\boldsymbol{p}$) of a nanowire under a sinusoidal AC electric field ($\boldsymbol{E})$ can be expressed as:

|  | $\boldsymbol{p=}\alpha\boldsymbol{E}$ | (S1) |
| --- | --- | --- |

where $\alpha$ is the polarizability matrix as a function of the nanowire’s orientation. If we extract the sinusoidal time-variant term and use the phasor expression:

|  | $\underline{\boldsymbol{p}}\boldsymbol{=}\alpha\underline{\boldsymbol{E}}$ | (S2) |
| --- | --- | --- |

where $\underline{\boldsymbol{p}}$and $\underline{\boldsymbol{E}}$ are vector of phasors. For simplicity, we decompose the vectors in the parallel and transverse direction of the nanowire’s axis to avoid the orientational dependence of the polarizability matrix. As a result, $\underline{\boldsymbol{p}_{\boldsymbol{\parallel}}}=\alpha_{\parallel}\underline{\boldsymbol{E}_{\boldsymbol{\parallel}}}$ and $\underline{\boldsymbol{p}_{\boldsymbol{\perp}}}=\alpha_{\perp}\underline{\boldsymbol{E}_{\boldsymbol{\perp}}}$, where $\underline{\boldsymbol{p}_{\boldsymbol{i}}}, \alpha_{i},\underline{\boldsymbol{E}_{\boldsymbol{i}}} \left( i=\parallel\text{or}\perp\right)$ are the dipole moment vector of phasor, polarizability, and electric field vector of phasor along the respective directions.

Our measurement of the rotation of a nanowire over time as it aligns to an electric field gives a discrete number of angles spaced at equal time intervals. The rotation speed over time is variable, however, so we cannot directly fit a line to the data to find a rate. Instead, recall from the equation (3) in the manuscript that the rotation of a nanowire over time is given analytically by

|  | $\frac{d\theta}{dt}=-A\sin\theta\cos\theta$ | (S3) |
| --- | --- | --- |

where $A$, the alignment rate, is a constant. Thus, $A$ may be approximated from the experimental data as:

|  | $A=\frac{-1}{t_{2}-t_{1}}\int_{\theta_{1}}^{\theta_{2}} \frac{d\theta}{\sin\theta\cos\theta}$ | (S4) |
| --- | --- | --- |

Note that as the nanowire approaches $\theta=0 and \pi/2$, the rotation speed approaches zero and equation. (S4) has non-integrable singularities. Also, Brownian motion dominates, adding noise to measurements. So, to ensure accuracy in our calculation of $A$ , we restrict data analysis to $\left| \theta_{1}-\pi/4 \right|<0.5$ and $\left| \theta_{2}-\pi/4 \right|<0.5$. We also require $\left| \theta_{1}-\theta_{2} \right|>0.5$ to avoid errors from too little data.

Regarding measurement: at low E-field frequencies, the alignment torque is much stronger than the torque at high frequencies. As a result, if the E-field strength were kept constant while measuring alignment rate at different frequencies, the nanowires would align either too fast at low frequencies or too slow at high frequencies for accurate analysis. Instead, using the parallel microelectrodes, we apply 15 V_pp_ when the torque is weak or 3 V_pp_ when the torque is strong, where torque strength is determined solely by observation from experiment (as it varies nonlinearly with frequency, laser intensity, etc.). In the data analysis, we normalize the alignment rates obtained at these two different voltages according to the square dependent of A to the electric field strength or voltage as$A=\frac{E_{0}^{2}}{2\gamma} {\mathrm{Re}(\alpha}_{\parallel}-\alpha_{\perp})$. The anisotropy of polarizability shown in Fig. 2a, b and 3b are the difference of real part polarizability between longitudinal and transverse direction ${\mathrm{Re}(\alpha}_{\parallel}-\alpha_{\perp})$. It is calculated from: ${\mathrm{Re}(\alpha}_{\parallel}-\alpha_{\perp})=\frac{2A\gamma}{E_{0}^{2}}$.

**Supplementary Note 4: Numerical Simulation Details**

The simulation is conducted in two parts. First, the Maxwell-Wagner polarization of the silicon nanowire in DI water is calculated by finite element analysis with the commercial software COMSOL. The model of the silicon nanowire consists of a bulky silicon part with a cylindrical shape of 500 nm in diameter and 10 $\mu m$in length and a thin oxidation shell of 1 nm in thickness surrounding it to account for the native oxidation layer in water. The calculation of the dipole moment is accomplished by the integration of the electrical potential along a circular loop. The integration approach for calculation of the dipole moment relies on the assumption of the orthogonality between the contributions of various orders of the electrical moment in contribution to the total electrical potential in an azimuthally symmetrical system. However, as mentioned in the text, the alignment behavior relies not only on the polarization in the long axial direction of the nanowire but also on that in the transverse direction. For the transverse polarization, the electric field is perpendicular to the long axis of the nanowire, and thus, the system does not have the azimuthal symmetry, and theoretically couldn’t be calculated from the integration methods. A first order approximation is adopted to overcome the issue that a large integration loop is used with a radius much larger than the dimension of the nanowire. By doing so, we are able to use the azimuthal symmetry in approximation for dipole moment calculation. To validate the approximation, we select two different integration loops, the first circular loop is in the same plane as the long axis of the nanowire, while the second circular loop is in the plane which is perpendicular to the long axis of the nanowire. The results of the dipole moment of from two different integration loops have only a minor difference less than 1%, which support the feasibility of approximation of azimuthal symmetry and the integration method. The parameters used in the simulation are listed in Table S1.

Supplementary Table S1. Simulation Parameters.

| Parameters | $\tau_{RC}$ | $\varepsilon_{si}/\varepsilon_{0}$ | $\varepsilon_{water}/\varepsilon_{0}$ | $\sigma_{water}$ |
| --- | --- | --- | --- | --- |
| Value | $5\times{10}^{-5}$s | 12 | 80.2 | $4\times{10}^{-4} S/m$ |

**Supplementary Note 5: Decomposed polarizability**

Here we show the simulation results of the respective contributions to the real part polarizability from the Maxwell-Wagner polarization and electrical double layer effect.


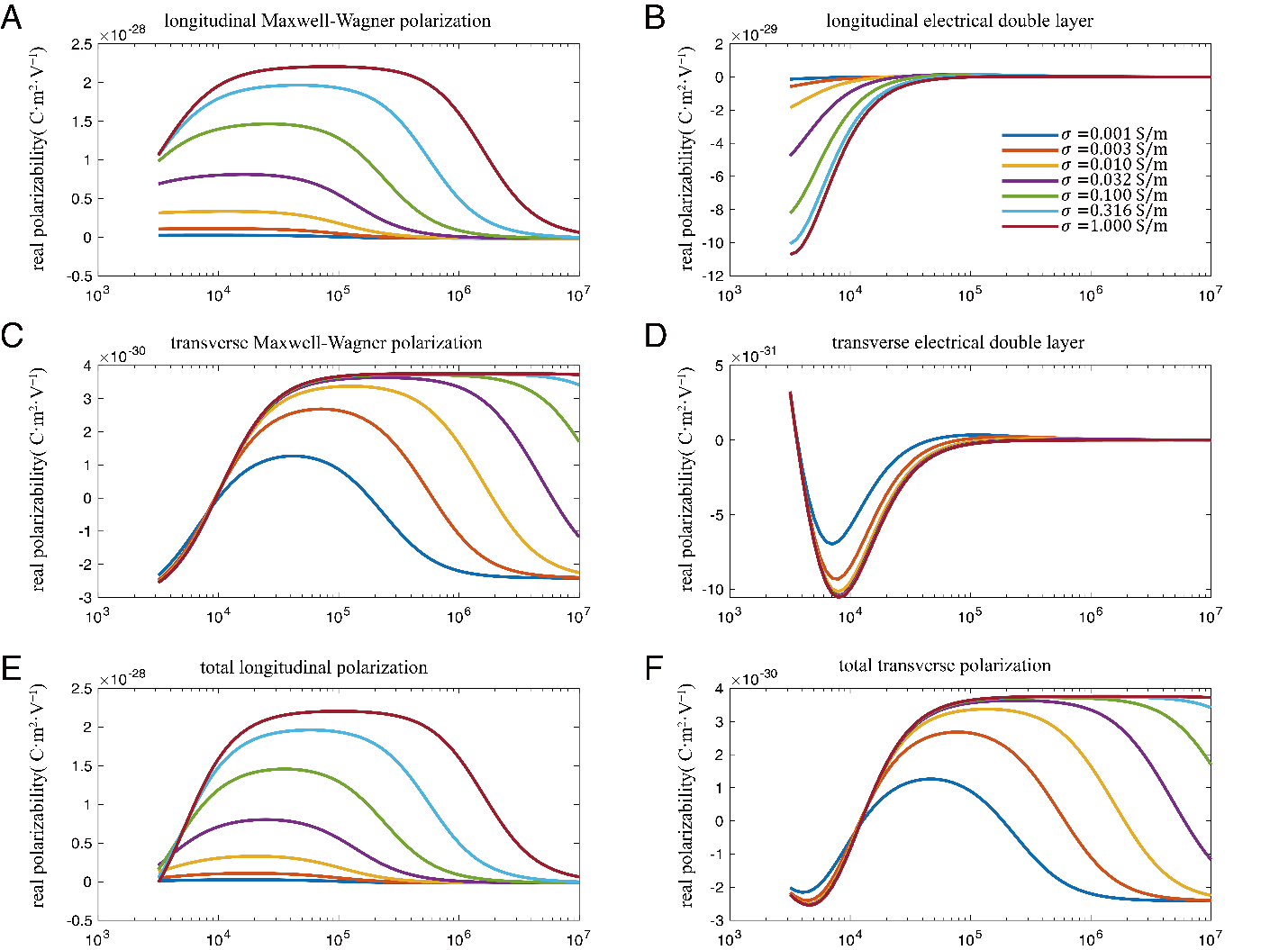


Supplementary Figure 1: Respective polarizability contribution of the Maxwell-Wagner polarization and the electrical double layer. (A, B) Real part polarizability in longitudinal direction of the nanowire from the Maxwell-Wagner polarization and electrical double layer, respectively. (C, D) Real part polarizability in transverse direction of the nanowire from the Maxwell-Wagner polarization and electrical double layer, respectively. (E, F) Combined real part polarizability from both effects in longitudinal and transverse directions of the nanowire, respectively.

**Supplementary Note 6: Oxidation effect**

We observed that nanowires stored in water after one day consistently align transversely to a 750 kHz electric field under background light, but there are less wires showing this behavior when tested immediately after being made. This might be due to surface oxidation of the nanowires or other reactions between Si and water. As discussed in our previous work, the surface states generated at the Si-SiO_2_ interface change the electrical properties of pure Si nanowires through both depletion and surface recombination processes.

The oxidation layer also plays a role with additional capacitance under AC electric field, which would influence the result of Maxwell-Wagner relaxation.

In experiment, we observed decrease in alignment rate when light intensity increases at low frequencies, *i.e.* 5 kHz, and the simulation result also shows decrease in alignment rate with increase of electrical conductivity in Fig. 3b (<5 kHz). First, as discussed in the manuscript, the alignment rate is directly proportional to the value of $Re(\alpha_{\parallel}-\alpha_{\perp})$, at low frequencies, $\alpha_{\perp}\ll\alpha_{\parallel}$, for simplicity, we only consider the polarization in the longitudinal direction of the nanowire ($\alpha_{\parallel}$). As shown in Supplementary Figure 2. we plot both the real and imaginary parts of the Maxwell-Wagner polarizability of the nanowire. If we model the nanowire made of pure silicon without surface oxidation, then the results are shown in Supplementary Figure. 2B, D. The real part of the polarizability increases as the light intensity increases, and the imaginary part of the polarizability is almost zero at low frequencies. However, once we include a thin oxide layer on the surface of the Si in the model, since Si always has a native oxidation layer, the simulation results as shown in Supplementary Figure 2A shows a decrease in the real-part polarizability at low frequency (<10 kHz). Meanwhile, the imaginary polarizability increases at low frequencies (Supplementary Figure 2C). After considering the effect of electrical double layer, the final results show decrease in real polarizability as electrical conductivity increases.

In a more intuitive picture, the thin oxide layer can be regarded as an additional capacitor placed in between the silicon nanowire and the electrical double layer. The dipole moment generated on the silicon nanowire first induces the polarization of the oxide capacitor, and then induces the EDL polarization. However, the polarization of oxide layer causes additional phase lag, as indicated in Supplementary Figure 2. The norm of the total polarization ($\sqrt{\mathrm{Re}\left( p \right)^{2}+Im\left( p \right)^{2}}$) still increases as the light intensity increases, but due to the existence of the oxide capacitance and the resulting additional phase lag, the imaginary part of the polarizability increases greatly, while the real part slightly decreases.


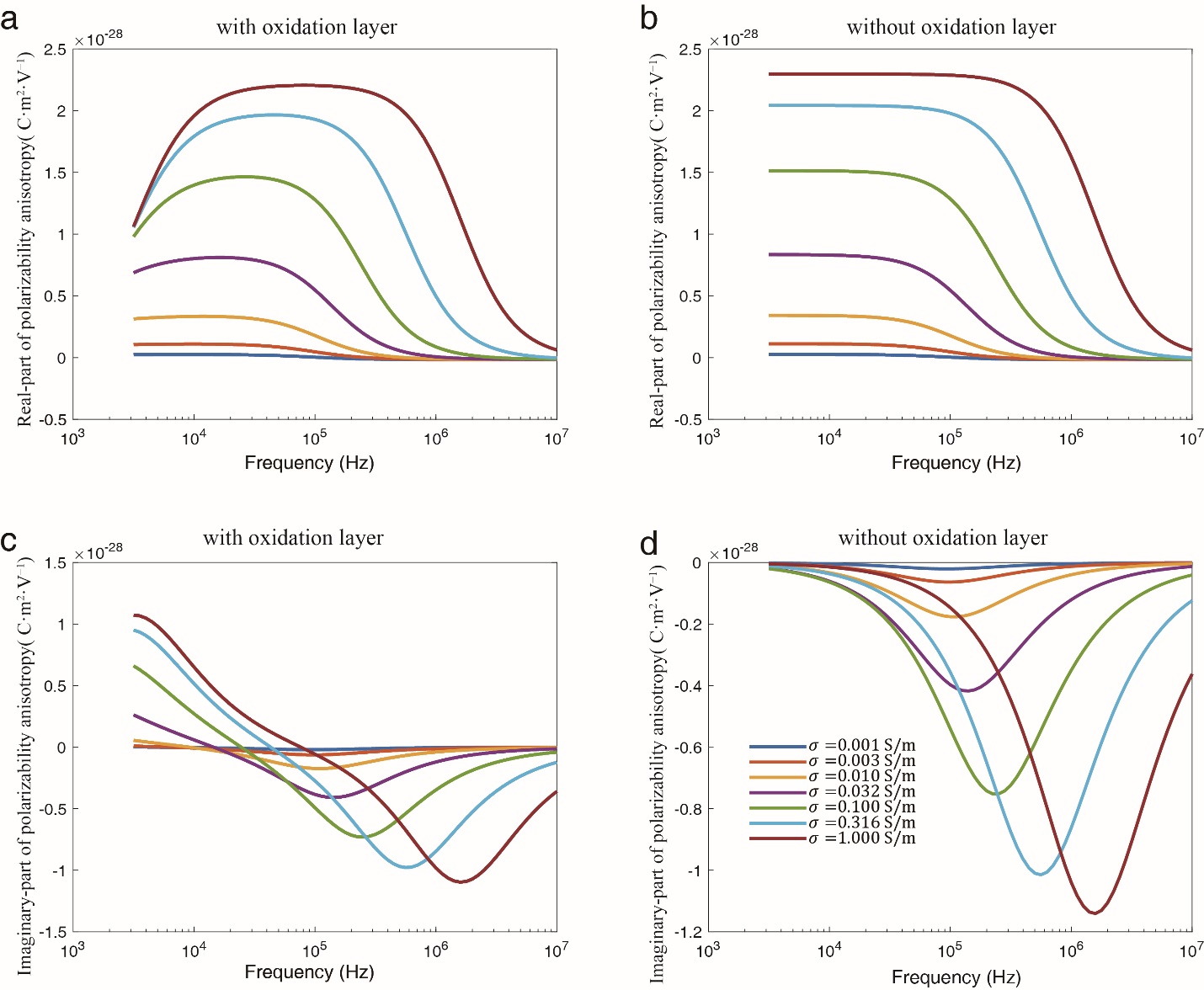


Supplementary Figure 2: Oxidation effect on the Maxwell-Wagner polarization. (A, B) Simulation results of real part polarizability anisotropy from the Maxwell-Wagner polarization with and without considering of a thin oxidation layer on the silicon nanowire surface. (C, D) The simulation results of imaginary part polarizability anisotropy from Maxwell-Wagner polarization with and without considering of a thin oxidation layer on the silicon nanowire surface.

**Supplementary Note 7: Out-Phase Oscillation**

**
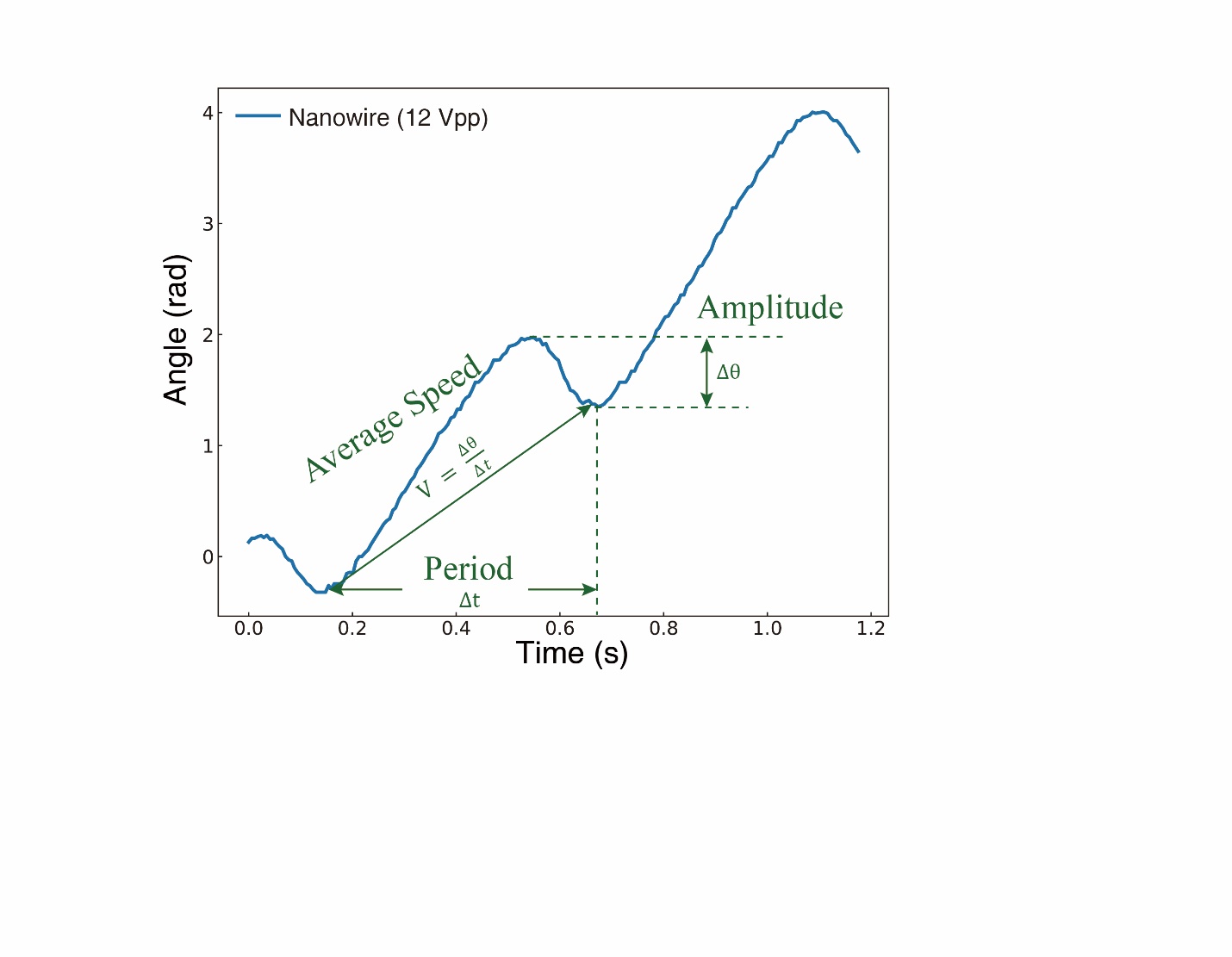
**

Supplementary Figure 3. Out-phase oscillation of the micromotor characterized by average speed, period, and amplitude.


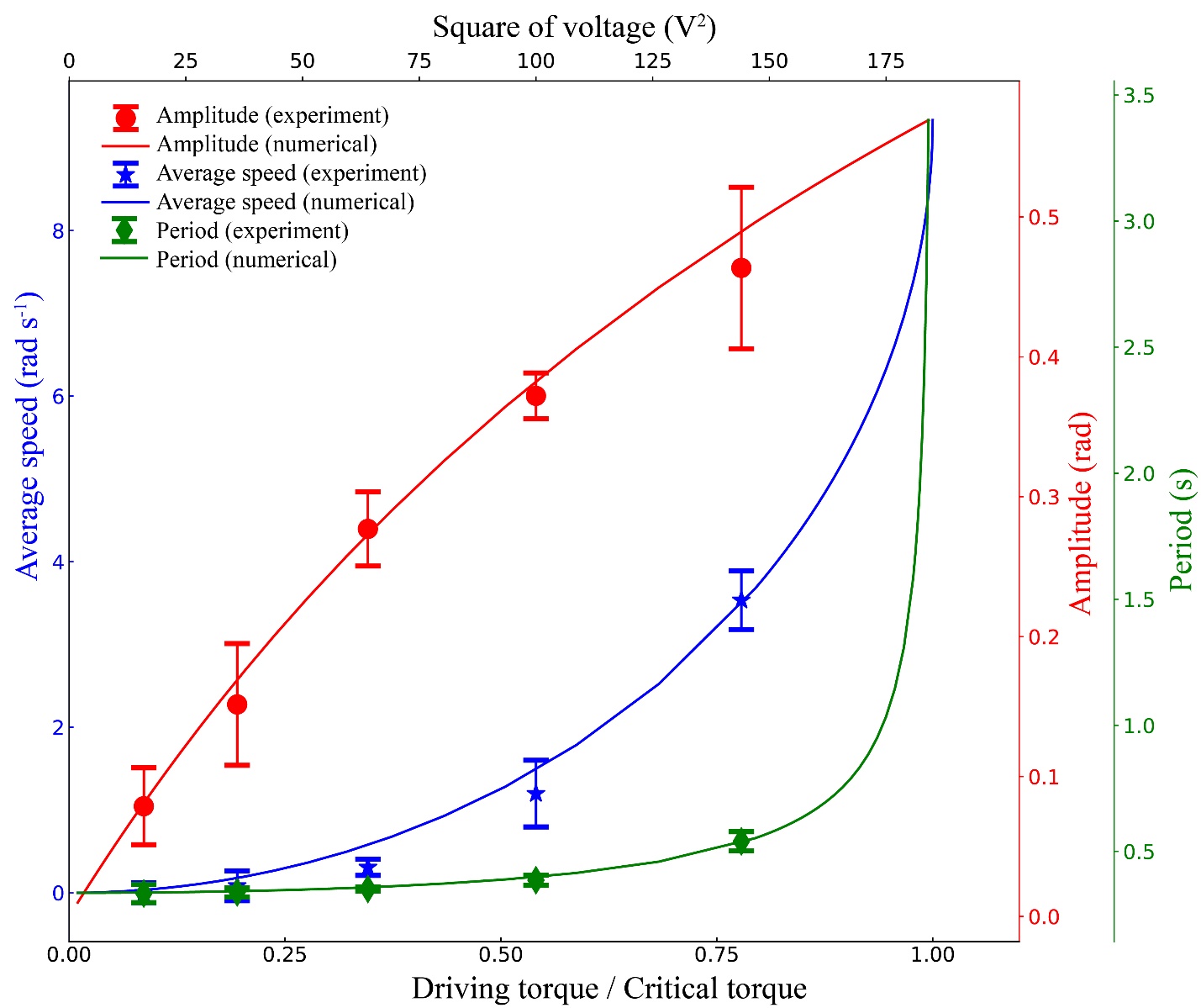


Supplementary Figure 4. Dynamic Analysis of the micromotor out-phase oscillation. The experimental data points of average speed, amplitude, and period are from the same data set as in Fig. 4c under 318 $mW/cm^{2}$ laser illuminance and with $\omega_{2}=1.49 \mathrm{Hz}$. The square of the critical voltage is fitted to be 185 $V^{2}$, and the corresponding driving torques are calculated in fraction of the critical torque. Numerical results shown in three curves are calculated with $\omega_{2}=1.49 \mathrm{Hz}$ at various driving torques excluding the influence of the Brownian motion.

When the motor is running out-phase, the driving torque needed to overcome the drag torque at the synchronous speed $\omega_{2}$ is greater than the maximum driving torque that the electric field can supply, so the nanowire lags behind the electric field. The nanowires oscillate slightly while still rotating slowly in the same direction of the rotating field as described in Fig. 4b. We define the amplitude, period and average speed of the out-phase oscillation for characterization (Supplementary Figure 3), and we further term the torque required to drive the motor at $\omega_{2}$ as critical torque ($\tau_{critical}=\gamma\omega_{2})$. To investigate the out-phase behaviors, we fix the synchronous speed of the field at $\omega_{2}=1.49 Hz$ and vary the voltage applied on electrodes within the out-phase mode and recording the amplitude, period and average speed of the oscillation. These values are plotted against the maximum driving torque from 1% to over 99% of the critical torque, where the motor behavior is in transition to in-phase mode as shown in Supplementary Figure 4. We also plot results of a numerical simulation for the same effect that agrees well with the experimental data.

The average speed increases monotonically from 0 to 9.42 rad/s (1.49 rps) as the driving torque increases up to the critical torque, above which the motor runs synchronous rotation at 9.42 rad/s. The oscillation amplitude also rises monotonically from 0 to ~ 0.57 rad as the driving torque increases and approaches to the critical torque. Once the driving torque equals to the critical torque, the amplitude of oscillation drops to zero without oscillation. Finally, the oscillation period increases slowly from ~0.33 s, about half of the electric field rotation period, until the driving torque is close to the critical torque, at which point the oscillation period rises asymptotically as the difference between motor rotation speed and field rotation speed approaches zero; when the driving torque is sufficient, the motor rotates in-phase with effectively infinite period.

**Supplementary Note 8: Characterization of the Synchronous Stepper Micromotor**

Since this novel motor capable of multiple operation modes with light control could be useful for micro/nanomachinery, it is desirable to quantitatively characterize its performance by using measures such as the pullout torque-speed curve, which is often used to evaluate comparable macroscopic stepper motors. To measure this curve, we measure the phase lag $\theta$, the angle between nanowire and electric field, by simultaneously recording the phase of the electric field at the custom function generator and the nanowire position via our computer vision tracking algorithm.


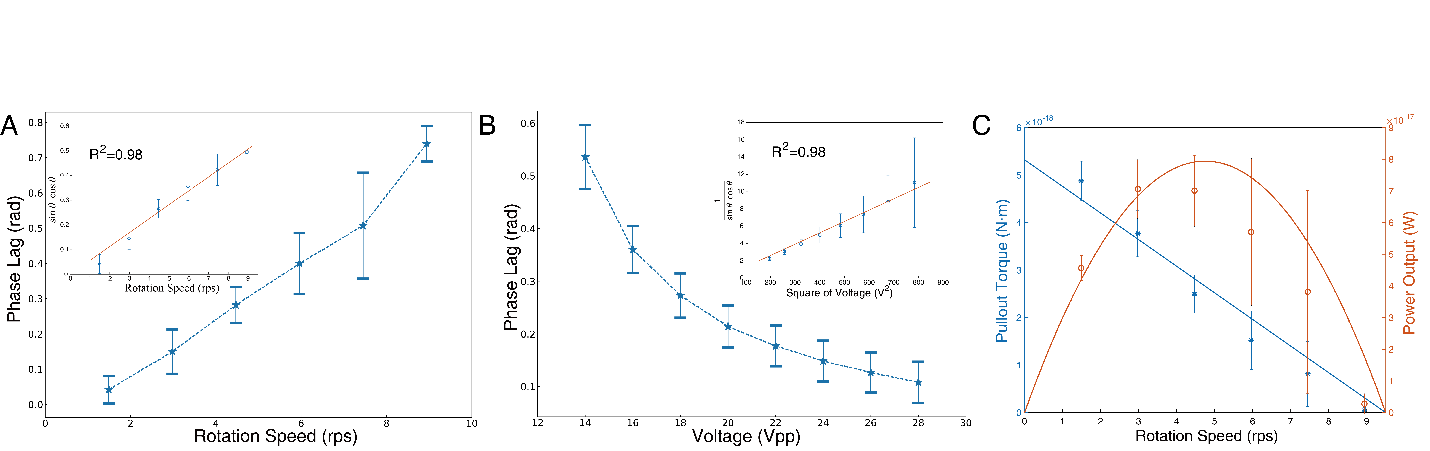


Supplementary Figure 5. Analysis of dynamics of a micromotor with in-phase rotation under 318 $mW/\mathrm{cm}^{2}$ laser illumination. (A) The phase lag $\theta$ as a function of the synchronous rotation speed $\omega_{2}$. Inset: The fitting between $\sin\theta\cos\theta$ and $\omega_{2}$ shows a high linear dependence with $R^{2}=0.98.$ (B) The phase lag $\theta$ as a function of the driving voltage. Inset: Fit between $\frac{1}{\sin\theta\cos\theta}$ and the square of driving voltage (V^2^) shows a good linear dependence with $R^{2}=0.98$. (C) Pull-out torque (in blue) and output power (in red) versus rotation speed of the micromotor at 15 V_pp_.

When the motor operates in phase, the driving torque balances the drag torque, as previously described, due to the low Reynold number of the system. Therefore, the phase lag $\theta$ is given by：

|  | $\sin\theta\cos\theta=-\frac{\omega_{2}}{A}=-\frac{{2\gamma\omega}_{2}}{E_{0}^{2}{\mathrm{Re}(\alpha}_{\parallel}-\alpha_{\perp})}$ | (S5) |
| --- | --- | --- |

This dependence can be verified by two experiments. Placing a nanowire in the quadruple microelectrodes, we first fix the amplitude of an applied electric field voltage at $15 V_{pp}$ and expose the nanowire to a 532-nm laser at $318 mW/\mathrm{cm}^{2}$ mW. We then measure the corresponding phase lag $\theta$ of the micromotor at different synchronous speeds $\omega_{2}$ , with results shown in Supplementary Figure 5A. We observe a linear dependence (inset of Supplementary Figure 5A) of $\omega_{2}$ on $\sin\theta\cos\theta$ with the coefficient of determination $R^{2}=0.98$ (with intercept forced at zero), confirming our understanding that the balanced driving and drag torques are proportional to the rotation speed $\omega_{2}$ as in equation (S5). Second, we fix the electric field rotation speed at $\omega_{2}=1.49 \mathrm{Hz}$, then measure the phase lag of in-phase rotation at different voltages (Supplementary Figure 5B). A linear dependence of ${1/V}_{0}^{2}$ on $\sin\theta\cos\theta$ is obtained with $R^{2}=0.98$ (with intercept forced at zero). This result further confirms that the driving torque is proportional to the square of the electric field intensity or voltage as also shown in equation (S5).

Another two key performance characteristics of industrial synchronous motors are the pull-out torque and output power, which represent the maximum torque and power that can be extracted from the motor before it loses in-phase synchronism. For our microscale stepper motors, the pull-out torque can be calculated from a given phase lag and speed in Supplementary Figure 5A as $\tau_{\mathrm{out}}=\left( 1-2\sin\theta\cos\theta\right)\tau_{max}.$ The output power at a given speed and voltage can be calculated as $P_{\mathrm{out}}=\tau_{\mathrm{out}}\cdot\omega=\left( 1-2\sin\theta\cos\theta\right)\tau_{max}\omega$ as shown in Supplementary Figure 5C. Here, we report normalized values to avoid dependence on $\gamma$, which cannot be easily calculated accurately because of a lack of good approximations for drag on nanowires rotating near a flat substrate.

(Assuming a data set has n values $y_{1},y_{2}, y_{3}, \ldots, y_{n},$ and are associated with a set of predicted value of $f_{1}, f_{2}, f_{3}, \ldots, f_{n}$.  $\bar{y}=\frac{1}{n}\sum_{i}^{n} y_{i}, SS_{tot}=\sum_{i} \left( y_{i}-\bar{y} \right)^{2}, SS_{res}=\sum_{i} \left( y_{i}-f_{i} \right)^{2}, and the$coefficient of determination $R^{2}\equiv1-\frac{SS_{res}}{SS_{tot}}$, where $SS_{res}$ is the residual sum of square, and $SS_{tot}$ is the total sum of square.)

**Supplementary Note 9: Reproducibility of Motor Performance**

The good reproducibility of motor performance is shown (Supplementary Figure 6) by the characterizations of 9 more motors in addition to the one shown in Fig. 4c in the main text. We also provide Supplementary Movie 9 to show the behaviors of multiple motors under a same stimulus.

**
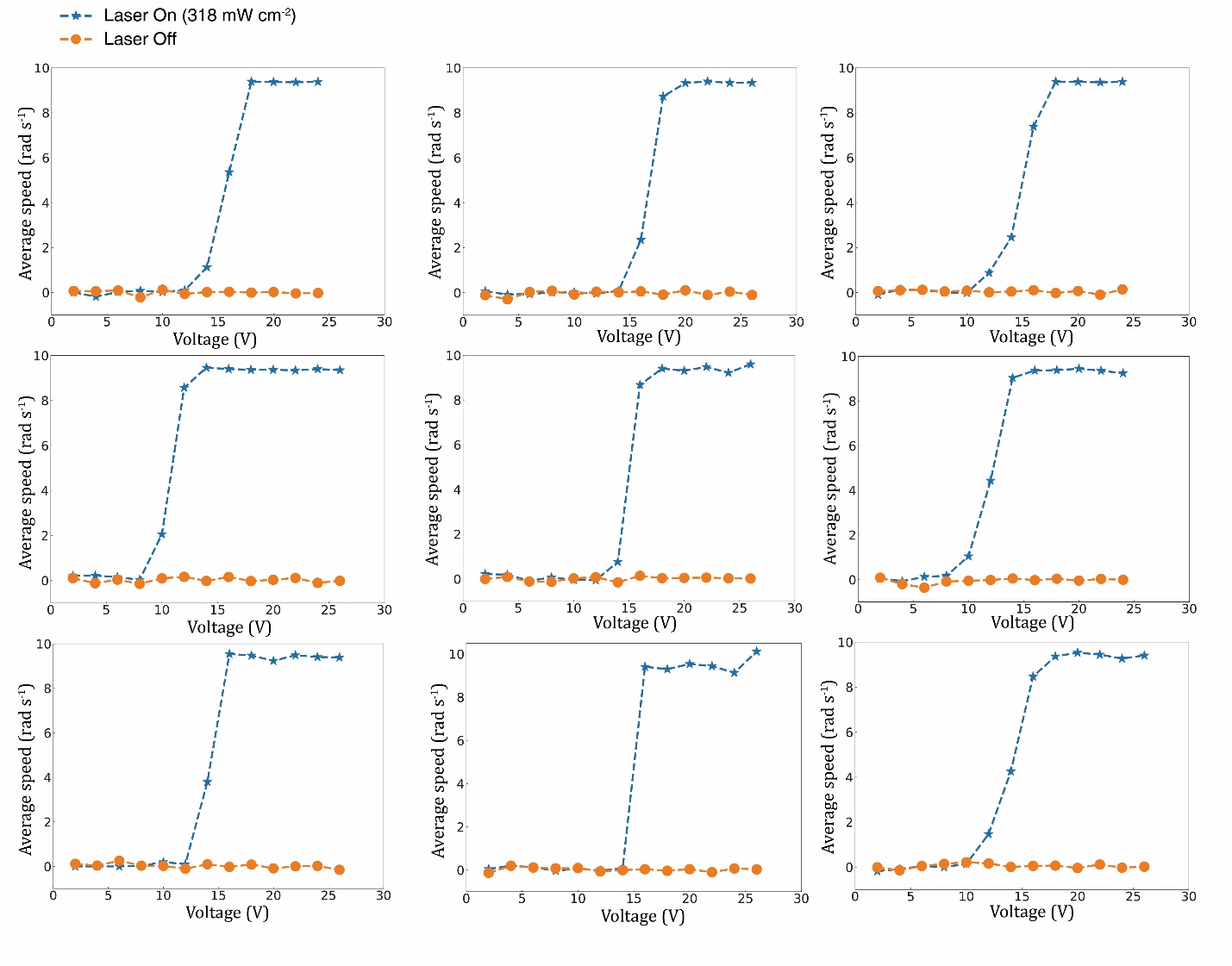
**

Supplementary Figure 6: Light switchable in-phase and out-phase rotation of a Si nanowire stepper motor. Average rotation speed of a micromotor versus driving voltage with (in blue) and without laser illumination (in orange). The driving electric field is 1.49 Hz, same as that the in-phase rotation of the nanowire.

**Supplementary Note 10: Lifetime characterization of the motors**

To characterize the lifetime of motors, we keep tracking the change of switching voltages above which the motor switches from out-phase oscillation to in-phase synchronous rotation using the same stimulus (318 $mW/cm^{2}$ 532-nm laser). The results of the switching voltage are shown in Supplementary Figure 7. In 8 days, the voltage gradually increases from 17 to 23 V, which indicates that the motor’s optical response and electrical polarizability decreases with time. Our current system has a maximum voltage output of 30 V. After day 8, we are not able to continue the measurement since some wires’ switching voltages exceed this voltage.

Overall, all the experimental results are obtained from motors freshly made within 2 days for consideration of reliability. In terms of device application, the lifetime of the motor is about 1 week.


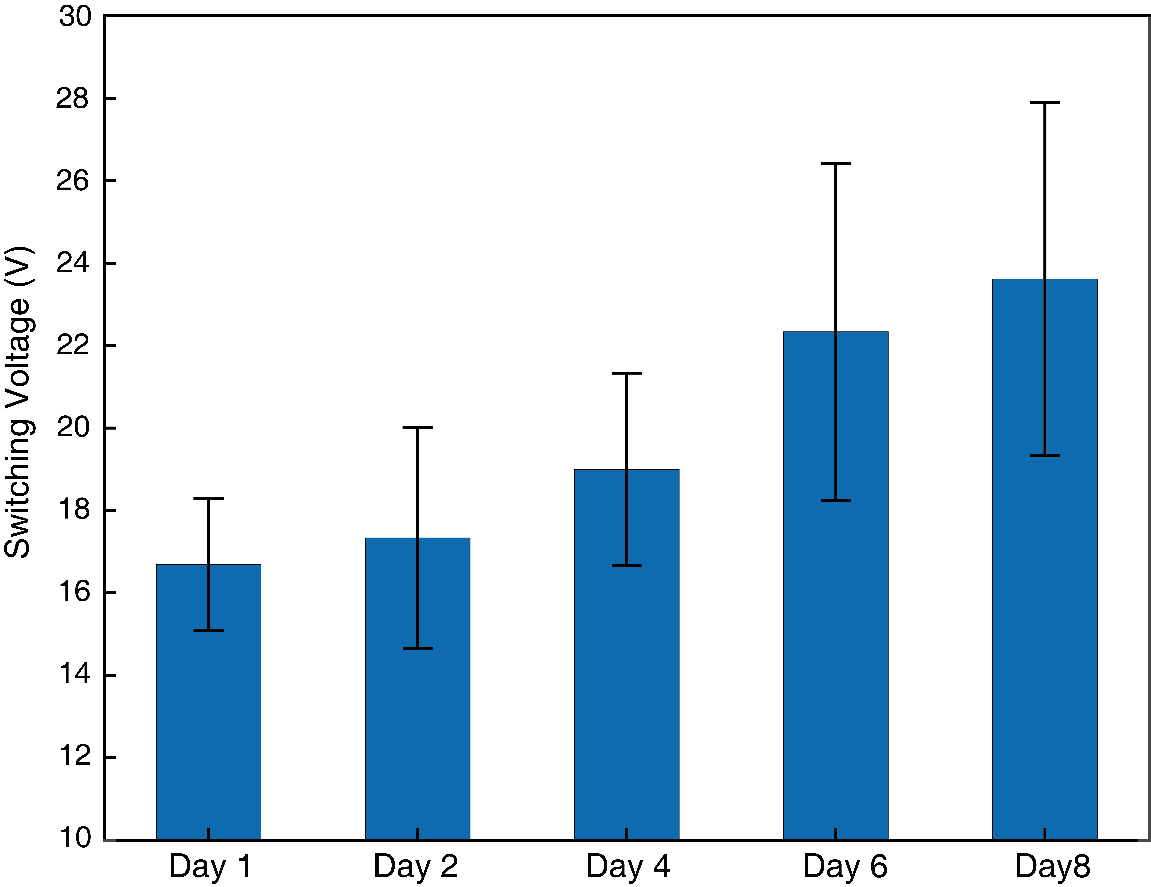


**Supplementary Figure 7:** Change of switching voltages of nanowires stored in DI water for different days. (error bars represent the standard deviation)

**Supplementary Note 11: Light Absorption and Photoconductivity**

When laser illuminates on a silicon nanowire (532 nm, higher energy than the bandgap of Si), electrons in the valence band absorb the energy of photons and get excited to the conduction band, leading to an increase of the total number of free electrons and holes. As a result, the electric conductivity of the nanowire increases. In an AC electric field, when the electrical conductivity ($\sigma)$ increases, the complex permittivity of the nanowire ($\varepsilon^{''}=\varepsilon-i\sigma/\omega$, where $\varepsilon,\sigma, and \omega$ are the real-part permittivity, electric conductivity, and electric-field frequency) will also change, and the total electric polarization in a suspension medium will be changed accordingly. Here we provide a more detailed discussion of the model:

1. Silicon nanowire light absorption:

First of all, for the same nanowire and same wavelength, the absorption cross-section can be considered as a constant within the linear optics regime (intensity of laser is moderate, without non-linear effects). Thus, the light power absorption is proportional to light intensity. Photons absorbed by the Si nanowire will generate pairs of electrons and holes, which drift under external electric fields, resulting in increase of electrical conductivity and electric dipole moment. The generation rate of electron-hold pairs is proportional to the light intensity within linear optics regime.

1. Light intensity and electrical conductivity^1^:

For a silicon nanowire of 500 nm diameter and $10 \mu m$length, under the illuminance of $318 mW/\mathrm{cm}^{2}$ at 532 nm, the total power absorbed is equivalent to the energy of $1.7\times{10}^{10}\mathrm{photons}s^{-1}.$ We adopt 50% as the quantum efficiency for a conservative magnitude estimation, as a result, the photo-excited carrier generation rate $\left( G \right) is 4.3\times{10}^{21}s^{-1} \mathrm{cm}^{-3}$. When the steady state of the system is reached, there must be a balance established between the photoexcitation and recombination, which indicates the recombination rate of the nanowire must equal to the carrier generation rate (G) of $4.3\times{10}^{21} s^{-1}cm^{-3}$. The recombination rate of excess carriers can be expressed as $\Delta n/\tau_{eff}$, where $\Delta n$ is the excess carrier density (for intrinsic silicon, $\Delta n=\Delta p$, and we need to just consider $\Delta n$ for the purpose of calculation), $\tau_{eff}$ is the effective excess carrier lifetime. For steady state, $G\cdot\tau_{eff}=\Delta n$. The effective lifetime is composed of two parts, the bulk lifetime and the surface lifetime, and can be expressed as $\frac{1}{\tau_{eff}}=\frac{1}{\tau_{bulk}}+\frac{4S}{d}$, where $\tau_{\mathrm{bulk}}$ is the bulky recombination lifetime mainly relating to Auger, Shockley-Read-Hall and radiative recombination, *S* is the surface recombination rate and *d* is the diameter of the nanowire. For nanowires, the bulk recombination is negligible, and the surface recombination dominates. After a comprehensive literature survey, we found that the surface recombination rate of silicon nanowire falls into the magnitude of ${10}^{4}\mathrm{cm}s^{-1}$ without special surface passivation^1-4^, and thus $\tau_{eff}\approx$ 5 ns for silicon nanowire of 500 nm diameter. The excess carrier density can then be calculated $\Delta n=G\cdot\tau_{\backslash eff}\approx2.2\times{10}^{13} cm^{-3}$. Therefore, we can calculate the photoconductivity given by $\Delta\sigma={(\mu}_{e}+\mu_{h})e\Delta n$ with a magnitude of ${10}^{-1} S m^{-1}$.

**Supplementary Note 12: Simulation of Electric Field Distribution between Quadruple Electrodes**

Here shows the simulation result of electric field distribution between quadruple electrodes with a gap of 500 $\mu m$ as Supplementary Figure 8. The color scale shows electrical potential distribution, and the lines represent electric fields.


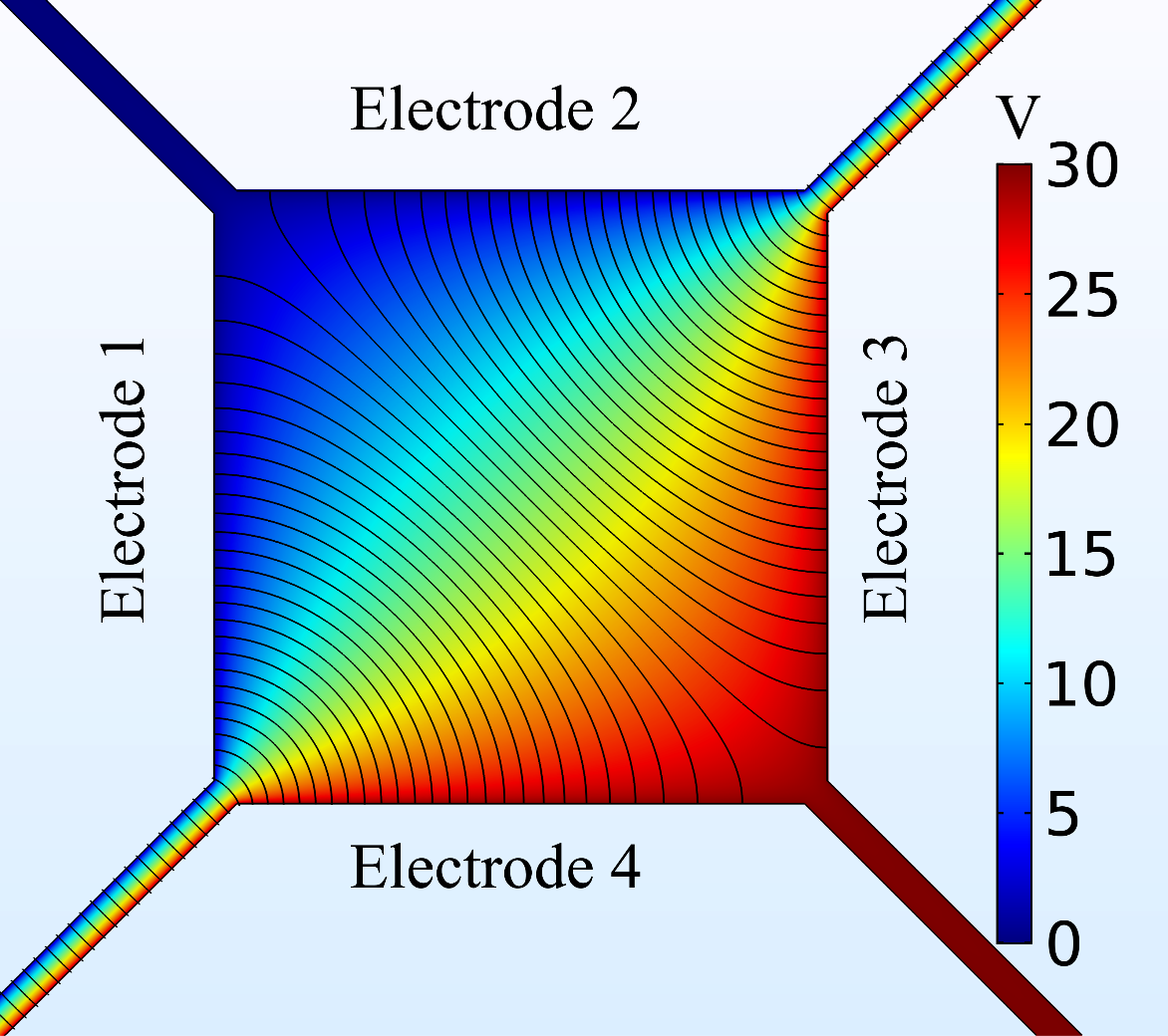


Supplementary Figure 8: Simulation result of electric field distribution in the center of a quadruple microelectrode when an E-field vector is at 45° with respect to the horizontal direction. The distribution of the electric field lines shows a much higher uniformity of the field at the center.

**References:**

1 Dan, Y. *et al.* Dramatic Reduction of Surface Recombination by in Situ Surface Passivation of Silicon Nanowires. *Nano Letters* **11**, 2527-2532, doi:10.1021/nl201179n (2011).

2 Demichel, O. *et al.* Surface recombination velocity measurements of efficiently passivated gold-catalyzed silicon nanowires by a new optical method. *Nano letters* **10**, 2323-2329 (2010).

3 Grumstrup, E. M. *et al.* Ultrafast carrier dynamics in individual silicon nanowires: Characterization of diameter-dependent carrier lifetime and surface recombination with pump–probe microscopy. *The Journal of Physical Chemistry C* **118**, 8634-8640 (2014).

4 Kato, S., Yamazaki, T., Kurokawa, Y., Miyajima, S. & Konagai, M. Influence of fabrication processes and annealing treatment on the minority carrier lifetime of silicon nanowire films. *Nanoscale research letters* **12**, 242 (2017).
