# Supplementary material for: Light programmable micro/nanomotors with optically tunable in-phase electric polarization
Source: Nat Commun. 2019 Nov 21;10:5275. doi: 10.1038/s41467-019-13255-6 (PMC6872749; doi:10.1038/s41467-019-13255-6)
Supplement: Supplementary file 3 — Description of additional supplementary files [file 41467_2019_13255_MOESM3_ESM.pdf]

Description of Additional Supplementary Information

File Name: Supplementary Movie 1

Description: This movie shows the setup of our custom software used to track nanowires. The user manipulates the greyscale threshold to give the computer vision tracking algorithm the best possible view of the nanowires.

File Name: Supplementary Movie 2

Description: Light readily switches the alignment direction of a nanowire to an electric field from perpendicular to parallel. Here, a nanowire is placed in the parallel microelectrodes, to which we applied a 15 Vpp and 750 kHz waveform. A laser beam of 111 mW/cm[illegible] is used to switch the nanowire alignment direction to be parallel to the field. When the laser is off, the nanowire returns to perpendicular alignment.

File Name: Supplementary Movie 3

Description: A nanowire rotates back and forth to signal "HELLO WORLD" in Morse code, demonstrating a unique method of communication. The nanowire is placed in the parallel microelectrodes with an electric field of 750 kHz at 25 Vpp such that it aligns perpendicular to the field. Laser light is toggled programmatically by a DMD to turn the nanowire towards the electric-field direction at the signaling times.

File Name: Supplementary Movie 4

Description:

Department of Mechanical Engineering · ETC II 5.160 · http://www.me.utexas.edu 1 University Station C2200 · Austin, Texas · 78712-0292 · (512) 471-1131 · Fax (512) 471-8727 COCKRELL SCHOOL OF ENGINEERING THE UNIVERSITY OF TEXAS AT AUSTIN

Rotation modes at different AC voltages with light. Here, a nanowire in the quadruple microelectrodes is driven by an electric field of [illegible] = 100 kHz rotating at [illegible] = 1.49 Hz, where the voltage applied to the microelectrodes begins with 12 Vpp and then changes to 20 Vpp. The nanowire is exposed to 318 mW/cm[illegible] laser.

File Name: Supplementary Movie 5

Description: A nanowire rotates continuously at synchronous speed in a sufficiently strong electric field. If the field is too weak, the nanowire experiences only out-phase rotation. Here, a nanowire is placed in an electric field of [illegible] = 100 kHz rotating at [illegible] = 1.5 Hz in the quadruple microelectrodes, and constantly exposed to 318 mW/cm[illegible] laser. The voltage applied to the microelectrode is scanned from 2 Vpp to 28 Vpp.

File Name: Supplementary Movie 6

Description: Light effect on the rotation of a nanowire at different AC frequencies. If the torque is low, the nanowire slips (out-phase rotation). Here, a nanowire (in the quadruple microelectrodes) is driven by an electric field of ◌ଵ = 100 kHz at 30 Vpp rotating at an assorted frequency of ◌ଶୀ from 1.5 Hz to 23.8 Hz. At each low rotation frequency, a laser of 318 mW/cmଶୀ is toggled to observe the difference on nanowire rotation (above 8.9 Hz, the nanowire cannot rotate when the laser is off).

File Name: Supplementary Movie 7

Description: A nanowire rotates synchronously with an electric field up to maximum speed, after which it exhibits out-phase rotation before stopping. Here, a nanowire is placed in a rotating electric field of ◌ଵ = 100 kHz at 30 Vpp in quadruple microelectrodes, and constantly exposed to laser light of 318 mW/cmଶୀ. The electric field rotation frequency ◌ଶୀ is scanned from 1.5 Hz to 26.8 Hz.

File Name: Supplementary Movie 8

Description: Demonstration of the utilization of light to control the operation mode of a nanomotor: a nanowire rotates continuously at synchronous speed with an electric field ("in-phase") under laser light, but barely twitches ("out-phase") without. Here, a nanowire (in the quadruple microelectrodes) is driven by an electric field of ◌ଵ = 100 kHz rotating at ◌ଶୀ = 2.98 Hz at 25 Vpp. A laser of 127 mW/cmଶୀ is toggled on and off to observe the nanowire switching rotation states.

File Name: Supplementary Movie 9

Description: Demonstration of the utilization of light to control the operation mode of multiple nanomotors. Multiple nanowires are driven by an electric field of ◌ଵ = 100 kHz rotating at ◌ଶୀ = 2.98 Hz at 30 Vpp. A laser of 318 mW/cmଶୀ is toggled on and off to control the switching of the rotation states of a large number of nanowires.

File Name: Supplementary Movie 10

Description: Individually controlled nanowire motors with DMD-generated laser spots. Here, a DMD generates two laser spots of 50 mW/cmଶୀ, which are each exposed onto one of two nanowires (in the quadruple microelectrodes). The nanowires are driven by an electric field of ◌ଵ = 100 kHz rotating at ◌ଶୀ = 1.49 Hz at 13 Vpp. The laser spots are then turned on and off independently, which can control a motor without affecting the other.

File Name: Supplementary Movie 11

Description: Operation of a nanowire to designated angular positions as a stepper motor. Here, the angle of a nanowire (in the quadruple microelectrodes) is controlled by tuning the direction of an electric field of 100 kHz at 10 Vpp under 127 mW/cm[illegible] laser.

File Name: Supplementary Data 1

Description: This data file ("Morse.dat") contains information of the position and angle of a nanowire versus time as it is stimulated by an intermittent laser to communicate words in Morse code (see Supplementary Movie 3). Interpret and plot this data to see the Morse code signal by placing this file in the same folder of the MATLAB script "interpreter.m" before running the script.

File Name: Supplementary Data 2

Description: This is a MATLAB script ("interpreter.m") that interprets and plots the nanowire motion data in "Morse.dat" to show its Morse code signal. Place the "morse.dat" file in the same folder before running this script.
